# Orbital abscess treated by ultrasound-guided fine needle aspiration and catheter drainage: A case report

**DOI:** 10.1097/MD.0000000000017365

**Published:** 2019-09-27

**Authors:** Yan Wang, Jie Zhang, Lei Dong, Hong Jiang, Xicheng Song

**Affiliations:** a Department of Otolaryngology–Head and Neck Surgery; b Department of Ultrasound, Qingdao University Medical College Affiliated Yantai Yuhuangding Hospital, Yantai, Shandong Province, China.

**Keywords:** acute sinusitis, catheter drainage, fine needle aspiration, orbital abscess, ultrasound-guided

## Abstract

**Rationale:**

Periorbital cellulitis or an orbital abscess caused by acute sinusitis is a serious acute infectious disease. If not treated in time, serious complications may occur.

**Patient concerns:**

A 16-year-old girl with a history of right-sided proptosis, periorbital swelling, chemosis, hypophasis, restricted ocular movement in the upward direction, and diminution of vision was referred to our institution. The clinic, computed tomography (CT) and magnetic resonance imaging (MRI) examination indicate right orbital abscess in the upper quadrant and sinusitis.

**Diagnoses:**

She was diagnosed with orbital abscess, acute sinusitis.

**Interventions:**

She underwent medical management, transnasal endoscopic surgery and then ultrasound-guided fine needle aspiration (FNA) and catheter drainage.

**Outcomes:**

She was completely cured without any complications or sequelae.

**Lessons:**

Performance of surgical drainage in a timely manner and administration of effective antibiotic treatment according to bacterial culture can reduce the complications of orbital abscesses. Ultrasound-guided FNA and catheter drainage is a safe, simple, and effective method for the treatment of orbital abscess.

## 1 Introduction

An orbital abscess is a serious infectious disease of the eye and may cause severe complications such as visual loss and death.^[1]^ Rahbar et al^[2]^ described the high risk of orbital cellulitis in the pre-antibiotic era, during which the rate of permanent loss of vision was 20% and the mortality rate associated with central nervous system complications was 17%. Even today, 15% to 30% of patients still develop visual sequelae. The etiology of an orbital abscess is often due to extensions of acute sinusitis.^[3]^ Timely and appropriate treatments can reduce the complications and mortality. Medical treatment is initiated, if no response, surgery is considered. The purposes of the surgery of orbital abscess are to reduce the pressure on the orbit, drainage of pus, and obtain pus for culture.^[4]^ The approaches of surgical drainage are the endoscopic approach, the eyelid approach. However, the upper eyelid approach may have complications or sequelae such as facial scars and eye movement disorders. It is difficult to adequately drain orbital abscess of the upper quadrant by transnasal endoscopic surgery. We herein report an upper quadrant orbital abscess in a patient who exhibited an unusual disease course and underwent a unique surgical drainage technique. To our knowledge, this is the only reported case of an orbital abscess treated by ultrasound-guided fine needle aspiration (FNA) and catheter drainage.

## 2 Case report

**Figure 1:**
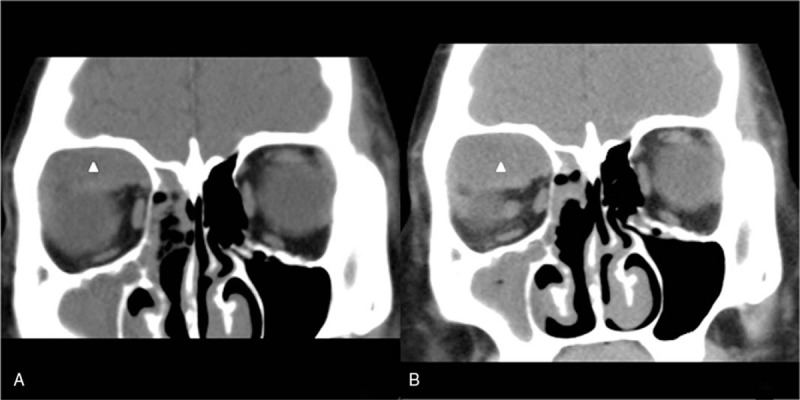
(A) Preoperative coronal computed tomography scan showing a right orbital subperiosteal abscess (white triangle). (B) Postoperative coronal computed tomography scan showing that the right orbital abscess (white triangle) is larger than that preoperatively.

A 16-year-old girl presented to our department with right nasal obstruction accompanied by a >1-month history of purulent discharge and a 4-day history of right-sided proptosis. About 1 month previously, she had developed an upper respiratory infection and presented with right nasal obstruction, purulent discharge, and frontal headache. She underwent medical management but showed no response to antibiotics. She later developed a fever, right-sided proptosis, periorbital swelling, chemosis, hypophasis, restricted ocular movement in the upward direction, and diminution of vision. Computed tomography showed right sinusitis and a soft tissue density in her right orbit (Fig. 1A). We administered intravenous sulbactam and a steroid and performed transnasal endoscopic surgery, including maxillary antrostomy, ethmoidectomy, frontal antrostomy, and decompression of the right orbit. On postoperative day 2, she had clinically improved and all of her symptoms had decreased. The culture of purulent discharge from the sinus showed normal flora.

**Figure 2:**
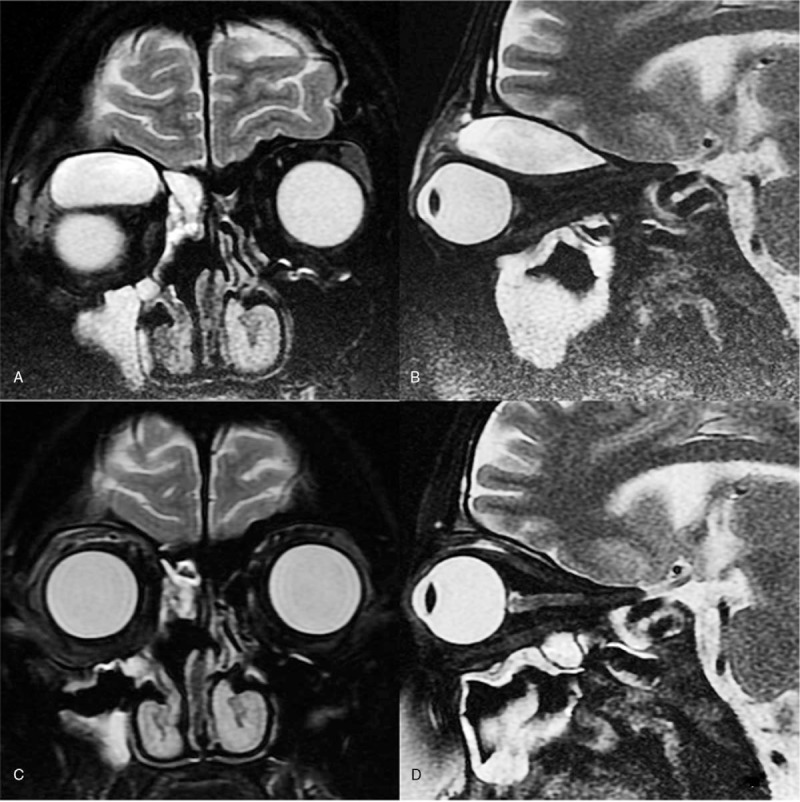
(A) Postoperative coronal T2-weighted magnetic resonance image showing that the right orbital abscess is pushing the eyeball forward and downward. (B) Postoperative sagittal T2-weighted magnetic resonance image showing that the right superior orbital abscess is pushing the eyeball forward and downward. (C) Coronal T2-weighted magnetic resonance image showing that the right orbital abscess has disappeared after ultrasound-guided fine needle aspiration and catheter drainage; the eyeball has returned to the normal position. (D) Sagittal T2-weighted magnetic resonance image showing that the right orbital abscess has disappeared after ultrasound-guided fine needle aspiration and catheter drainage; the eyeball has returned to the normal position.

**Figure 3:**
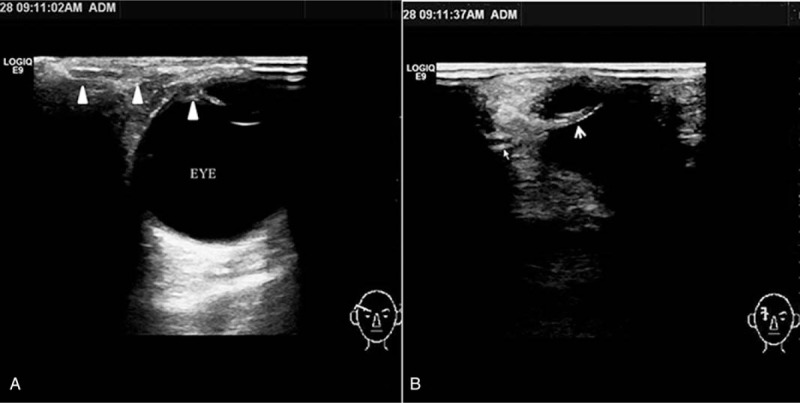
(A) Ultrasound showing the position of the drainage catheter (white triangle) in the horizontal direction. (B) Ultrasound showing the position of the drainage catheter (white arrow) in the vertical direction.

However, on postoperative day 12, her condition deteriorated again with increased periorbital swelling and downward shifting of the right eyeball. Computed tomography and magnetic resonance imaging revealed an upper quadrant orbital abscess that was pushing her right eyeball forward and downward (Figs. 1B and 2A, B). We consulted with the ophthalmology department, who suggested surgical drainage by an upper eyelid approach. We also suggested that she undergo an eye ultrasound and told her and her parents that ultrasound-guided FNA with catheter drainage has been widely used in the treatment of abscesses in the thyroid, neck, and other regions in our experience. It has the advantages of high safety, few complications, small scars, and no affect on the appearance. To avoid the possible complications of ptosis and cicatrization, the patient and her parents accepted this treatment method (Fig. 3). The sonographer placed the drainage catheter under ultrasound guidance and local anesthesia. Detailed steps were as follows: ultrasound examination confirmed the needlepoint next to the right eyelid, 2% lidocaine local infiltration anesthesia, under the ultrasound guiding the puncture was performed with a 6F pigtail drainage catheter set (one step type), puncture needle tip straight into the abscess cavity, pulled out the needle core, yellow pus flowed out, and 5 mL of purulent material was obtained for bacterial culture, then the drainage bag was connected, and the drainage sleeve was fixed by adhesive plaster.

We irrigated the drainage catheter with normal saline twice a day and changed the antibiotic to vancomycin according to the advice of an infectious disease specialist. On the third day after catheter drainage, she had remarkably improved and underwent another ultrasound examination. The orbital abscess had disappeared and the drainage catheter was removed. The culture of the abscess contents revealed *Streptococcus intermedius*, and vancomycin was one of the sensitive antibiotics. After a 1-week course of intravenous vancomycin, another magnetic resonance imaging scan showed no orbital abscess (Fig. 2C, D). According to the bacteriological culture and de-escalation antibiotic therapy, we changed the antibiotic to penicillin. Three days later, the patient was discharged home without visual damage or any other sequelae. The patient was followed up >3 years and no sequelae.

## 3 Discussion

Orbital cellulitis or abscess formation is a serious infection that can cause severe complications such as blindness, cavernous sinus thrombosis, meningitis, subdural empyema, and brain abscess.^[5]^ Orbital abscesses may result from acute sinusitis, trauma, insect bites, conjunctivitis, dacryocystitis, blepharitis, and implants.^[6–8]^ Imaging examination is essential because it can show the location and extent of the lesions.

Bacterial cultures reveal typical upper respiratory tract pathogens seen in cases of acute sinusitis, including *Streptococcus pneumoniae*, *Haemophilus influenzae*, *Moraxella catarrhalis*, *Streptococcus pyogenes*, *Staphylococcus aureus*, α-hemolytic and non-hemolytic streptococci, and anaerobic bacteria.^[5]^ Although the patient in the present case had undergone surgical drainage, the first culture had failed to detect bacteria, and the initial empirical antibiotic therapy did not produce expected relief, even leading to worsening of the patient's condition.

Surgical drainage performed in a timely manner might prevent the complications of vision loss. The purposes of surgical drainage are to lower the orbital pressure, drain the abscess, and obtain a specimen for culture.^[4]^ One of the absolute indications for endoscopic sinus surgery in children is acute sinusitis complicated by a suborbital periosteal abscess or orbital abscess.^[9]^ To our knowledge, the most common treatment method is endoscopic surgery, and the next most common is surgery performed by a peripheral orbital approach. In this case, we adopted a new technique of ultrasound-guided FNA and catheter drainage (Fig. 3). This is a novel orbital abscess drainage technique, although it has been used for drainage of liver abscesses,^[10]^ biliary drainage,^[11]^ pancreas tail cysts,^[12]^ intra-abdominal abscesses,^[13]^ and thyroid abscesses.^[14]^ In 1985, Herzon^[15]^ reported the use of FNA in severe abscesses in head and neck. However, 2 cases (2/10, 20%) required incision and drainage because they only performed puncture drainage and did not connect the continuous drainage device. Ilyin et al^[16]^ reported that 2 cases of thyroid abscess were treated with needle aspiration twice and after puncture drainage antibiotics were injected into the abscess cavity. Then, Yildar et al^[14]^ reported 1 case of acute suppurative thyroiditis who was successfully treated by sonographically guided percutaneous pigtail drainage catheter. This technique has been widely used for abscess drainage, the orbital abscess in the upper quadrant and avoiding facial scars, so we took this way.

We irrigated the drainage catheter with normal saline twice a day, and to avoid breaking the capsule of the abscess, we followed the principle of “the injected is less than the drained.” Our experience has shown that ultrasound-guided FNA is a minimally invasive, rapid, and efficient way to treat superior orbital abscesses. It has the advantages of minimal intraoperative complications, no surgical incision, rapid healing, and no scarring.

## 4 Conclusion

The report has introduced the application of ultrasound-guided FNA for treatment of an upper quadrant orbital abscess and has highlighted the importance of effective antibiotic treatment. Performance of surgical drainage in a timely manner and administration of effective antibiotic treatment according to bacterial culture can reduce the complications of orbital abscesses.

## 5 Ethics

This case report has gotten the informed consent of patient and her father during treatment and for publication.

## Acknowledgments

The authors would like to show their deepest gratitude to their director of otolaryngology, Dr Song Xicheng, a respectable, responsible, and resourceful scholar, who has provided them with valuable guidance at the writing of this thesis.

This paper has NOT been published elsewhere. All authors have read and approved the content, and agree to submit for consideration for publication in the journal.

## Author contributions

**Conceptualization:** Xicheng Song.

**Data curation:** Yan Wang.

**Formal analysis:** Yan Wang.

**Funding acquisition:** Yan Wang.

**Supervision:** Jie Zhang, Lei Dong, Hong Jiang.

**Writing – original draft:** Yan Wang.

**Writing – review & editing:** Xicheng Song.
